# Local adiposity may be a more reliable predictor for infection than body mass index following total knee arthroplasty: a systematic review

**DOI:** 10.1186/s40634-023-00680-2

**Published:** 2023-11-06

**Authors:** John J. Heifner, Philip A. Sakalian, Robert J. Rowland, Arturo Corces

**Affiliations:** 1 Miami Orthopaedic Research Foundation, 11801 SW 90th Street Suite 201, Miami, FL 33186 USA; 2 Department of Orthopaedic Surgery, Larkin Hospital, Coral Gables, FL USA

**Keywords:** Adiposity, BMI, Fat thickness, Periprosthetic joint Infection, Revision TKA, Soft tissue thickness

## Abstract

**Purpose:**

Improved understanding of the factors that predispose TKA patients to infection has considerable economic and medical impact. BMI is commonly used as a proxy for obesity to determine the risk of postoperative infection. However, this metric appears to be fraught with inconsistency in this application. BMI is a simple calculation which provides general insight into body habitus. But it fails to account for anatomic distribution of adipose tissue and the proportion of the mass that is skeletal muscle. Our objective was to review the literature to determine if local adiposity was more predictive than BMI for infection following TKA.

**Methods:**

A database search was performed for the following PICO (Population, Intervention, Comparison, and Outcome) characteristics: local measurements of adiposity (defined as soft tissue thickness or fat thickness or soft tissue envelope at the knee) in patients over 18 years of age treated with total knee arthroplasty used to determine the relationship between local adiposity and the risk of infection (defined as prosthetic joint infection or wound complication or surgical site infection).

Quality was assessed using the GRADE framework and bias was assessed using ROBINS-I .

**Results:**

Six articles (*N*=7081) met the inclusion criteria. Four of the six articles determined that adiposity was more associated with or was a better predictor for infection risk than BMI. One of the six articles concluded that increased adiposity was protective for short term infection and that BMI was not associated with the outcome of interest. One of the six articles determined that BMI was more strongly associated with PJI risk than soft tissue thickness.

**Conclusion:**

The use of adiposity as a proxy for obesity in preoperative evaluation of TKA patients is an emerging concept. Although limited by heterogeneity, the current literature suggests that local adiposity may be a more reliable predictor for infection than BMI following primary TKA.

**Level of evidence:**

IV systematic review

## Introduction

Infection is a well-documented complication following total knee arthroplasty (TKA) with considerable morbidity to the patient and burden to the health care system [2, 15, 16, 21]. Although the aggregate incidence of prosthetic joint infection (PJI) following TKA remains stable, there is expectation for increasing volume of post-TKA infection given the projections for increasing rates of primary TKA [1, 4, 14, 18, 20].

Body mass index (BMI in kilograms/meters^2^) is a simple calculation which provides general insight into body habitus. However, it fails to account for the anatomic distribution of adipose tissue and the proportion of the mass that is skeletal muscle. Further, the anatomic distribution of adipose has been shown to be an important variable in associating obesity with comorbid conditions [6, 8, 29]. Despite being commonly utilized as a predictor for infection following TKA, BMI has demonstrated inconsistency in this application [19, 24, 27]. Recent practice has restricted access to primary TKA based on obesity [5, 13]. Often these restrictions utilize a maximum BMI value [17]. Whether this practice is reasonable based on the efficacy of BMI is debated [11].

Recent investigations indicate that local adiposity is gaining momentum as a reliable predictor for postoperative infection. Subcutaneous fat thickness has been associated with increased rates of infection following abdominal surgery [22], spine surgery [7], hip fracture surgery [9], and colorectal surgery [10]. Therefore, it is a reasonable assessment that the same physiologic and mechanical factors which predispose to infection in these procedures may further the risk of infection following TKA. The local adipose-related variables which may contribute to infection include lengthened operative time, increased insult due to retraction and dissection and the metabolic and immune related compromise that are inherent to adipose tissue. Given the expansion of recent investigations, a comprehensive aggregation of evidence is needed to better understand the predictive capacity of local adiposity for infection following TKA.

Our objective was to review the literature to determine if local adiposity was more predictive for infection than BMI following primary TKA.

## Materials and methods

### Search protocol

A database search was performed in Google Scholar and PubMed on September 28, 2023, in keeping with the Preferred Reporting Items for Systematic Reviews and Meta-Analyses (PRISMA) guidelines. Keywords utilized were “soft tissue”, “adipose”, “adiposity”, “fat”, “obesity” AND “TKA/knee arthroplasty/knee replacement”. Duplicate and irrelevant articles were identified by title and author list.

### Guidelines for study inclusion

The Population, Intervention, Comparison, and Outcome characteristics (PICO) were the following: local measurement of adiposity (defined as soft tissue thickness or fat thickness or soft tissue envelope) in patients over 18 years of age treated with primary total knee arthroplasty used to determine the relationship between local adiposity and the risk of infection (defined as prosthetic joint infection or wound complication or surgical site infection or superficial/deep infection).

P = TKA > 18yo.

I = local measurement of adiposity.

C = adiposity and BMI.

O = risk of infection.

Exclusion criteria were articles that evaluated the risk of infection following revision TKA, and articles that did not describe the parameters for measuring local adiposity. Articles that described systemic adipose (body fat percentage) were excluded.

### Risk of bias assessment

The Cochrane ROBINS-I (risk of bias in non-randomized studies of interventions) was used to assess the risk of bias for each included article. The following domains were compiled: confounding, selection of participants, classification of interventions, deviation from intended interventions, missing data, measurement of outcomes, and selection of reported result. For each domain, a low, moderate, or serious level of risk was selected. For each article, the overall level of risk equated to the highest level of risk across the domains.

### Quality assessment

The GRADE (Grading of Recommendations, Assessment, Development, and Evaluation) framework was used to assess the quality of the evidence. Determining the phase of investigation was the starting point for the quality evaluation. The following factors were used to downgrade the presenting evidence: limitations, inconsistency, indirectness, imprecision, and publication bias.

### Data extraction

The following variables were collected: study methodology, the specific parameters for measuring adiposity and the data for postoperative infection. Articles were grouped based on the reported method of measuring adiposity. One group measured adiposity on lateral radiograph. The other group measured adiposity on lateral and anteroposterior radiograph. Statistical analyses were unable to be performed due to the methodological heterogeneity of the included articles. Thus, the current work is analytical in nature without aggregate quantified outcomes.

## Results

**Fig. 1:**
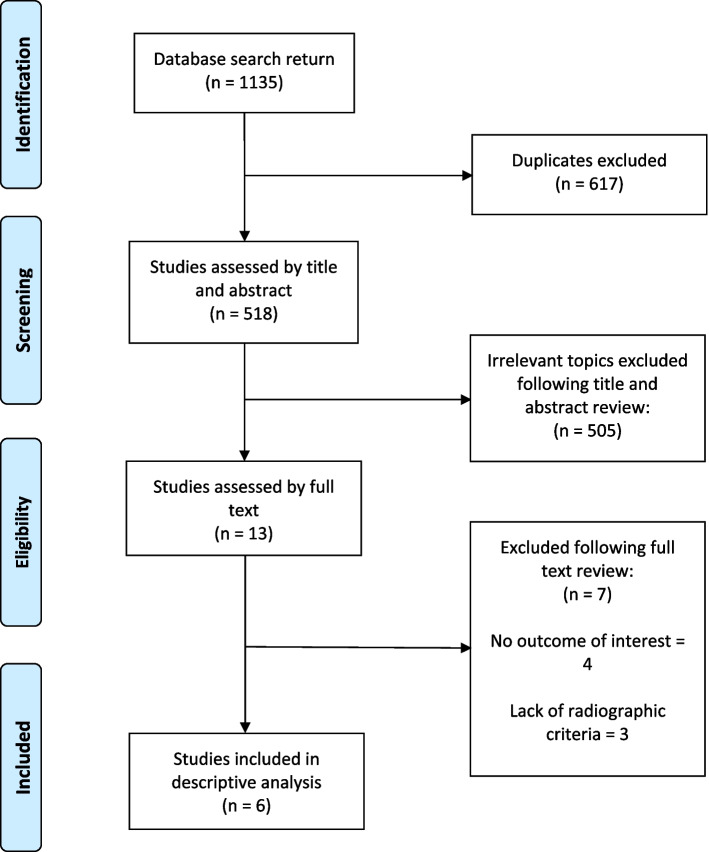
The sequence of database search and identification of included and excluded articles

Six articles (*N* = 7081) met the inclusion criteria following assessment of 518 by title and abstract (Fig. 1). There were no criteria disagreements between authors regarding inclusions and exclusions.

All included articles had at least one domain that was identified as having a moderate risk of bias (Table 1). This indicates that none of the articles can be considered comparative to a well-executed randomized trial, per the established interpretations. None of the articles contained domains identified as having a critical risk of bias.

**Table 1:**
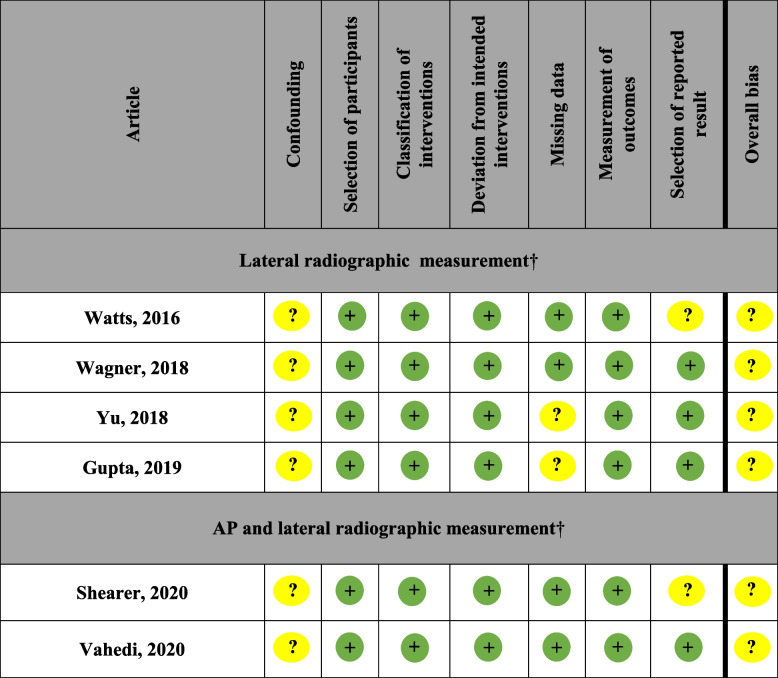
Cochrane risk of bias ROBINS-I (risk of bias in non-randomized studies of interventions) for included articles with green indicating low risk, yellow indicating moderate risks, and red indicating serious risk [12, 19, 23, 24, 27, 28]

†Radiographic measurement of adipose/soft tissue, anteroposterior (AP)

Within the GRADE framework, all included articles were classified as phase I investigations which indicates an emerging topic with weak evidence (Table 2). The outcomes were grouped according to the following radiographic methods of local adipose measurement: on anteroposterior radiograph, and on anteroposterior and lateral radiograph. All groups demonstrated limitations for inconsistency and publication bias.

**Table 2:** An adapted Grading of Recommendations Assessment, Development and Evaluation (GRADE) summarization for a systematic review of local adiposity as a predictor for infection following TKA

Outcome	Phase of investigation	Limitations	Inconsistency	Indirectness	Imprecision	Publication bias
Anteroposterior radiograph^a^	Explanatory (phase I)	✗	✓	✗	✗	✓
Anteroposterior and lateral radiograph^a^	Explanatory (phase I)	✗	✓	✗	✗	✓

^a^soft tissue or fat thickness/adiposity/body fat measurements, ✓ - serious limitations, ✗- no serious limitations

Four articles measure adiposity on lateral radiograph, and two measured adiposity on anteroposterior and lateral radiograph (Table 3). Two of the six articles evaluated infection at one year postoperatively [19, 28] and one of six evaluated infection at a minimum of two years postoperatively [23]. Three of the six reported 90-day data on reoperation for wound complication or infection [27], and surgical site infection (SSI) [12].

**Table 3:** Literature review for radiographic measure of local adiposity as a predictor for infection following TKA

Study	N	Follow up^a^	Outcome^a^	Femur^b^	Patella^b^	Tibia^b^	Findings
Lateral radiograph							
Watts, 2016 [27]	116	5 years (mean)	90-day reoperation for wound complication/ infection in morbid obesity		Fat thickness at the midpatella	Fat thickness at the most prominent aspect of tibial tubercle	Fat thickness was associated with a significantly increased risk of reoperation
Wagner, 2018 [24]	528	NR	SSI		Fat thickness at the midpoint of the patella		Fat thickness was a significant predictor of SSI; BMI was not
Yu, 2018 [28]	374	1 year	Wound complications @ 1 year minimum	Soft tissue width colinear to a line connecting the superior patella to the superior posterior condyle		Soft tissue width colinear to a line across the tibial plateau	Increased soft tissue envelope size was associated with an increased risk of wound complication; BMI did not have this association
Gupta, 2019 [12]	494	NR	90-day SSI in non-morbid obesity		Fat thickness at the midpatella	Fat thickness at the most prominent aspect of tibial tubercle	BMI not associated with 90-day SSI, increased patellar fat thickness was protective for developing SSI
**Anteroposterior and lateral radiograph**							
Shearer, 2020 [19]	4745	NR	PJI @ 1 year		Ratio of the fat at the midpoint of the patella to the length of the patellar articular surface	Ratio of width of the plateau to the width of the leg	BMI was a better predictor of PJI than local adiposity
Vahedi, 2020 [23]	824	5.7 years (mean)	PJI @ 2 year minimum	Soft tissue thickness from the quad tendon to the skin, 8 cm superior to the joint		Soft tissue thickness from the medial aspect of the tibial plateau to the skin	Increased soft tissue thickness was significantly associated with increased risk of PJI, independent of BMI

^a^*NR* not reported, *PJI* periprosthetic joint infection, *SSI* surgical site infection, *cm* centimeters

^b^Radiographic measurement of adipose/soft tissue

Four of the six articles determined that adiposity was more associated with or was a better predictor for infection risk than BMI [23, 24, 27, 28]. These findings were significant in three of the six articles [23, 24, 27]. One of the six articles concluded that increased adiposity was protective for short term infection and that BMI was not associated with the outcome of interest [12]. One of the six articles determined that BMI was more strongly associated with PJI risk than soft tissue thickness [19].

## Discussion

The evaluation of adiposity as a predictor for infection following TKA is an emerging concept as evidenced by five of the six reviewed articles being published within the last five years. The current results demonstrate that local adiposity may be a more reliable predictor than BMI for infection following TKA. These findings occurred across multiple methodologies and adiposity measurements including prepatellar, pretubercular, medial joint line and anterior femoral. Within some reports, these findings were statistically significant [23, 24, 27]. Vahedi et al. (Vahedi) demonstrated that increased soft tissue thickness was significantly associated with increased risk of prosthetic joint infection (PJI) at a minimum of two years postoperatively. This finding was independent of BMI levels. In contrast, the report by Shearer et al. [19] across a large sample (62% of the collective N) reinforce the already prevalent use of BMI as a predictor for postoperative infection. The authors concluded that BMI was more predictive for PJI at one year postoperatively than local adiposity. Notably, Gupta et al. [12] demonstrated that increased fat thickness may be protective against infection. This finding was postulated to be due to the vascular anatomy which may remain intact with an appreciable depth of subcutaneous adipose.

Recent reports estimate that nearly 50% of adults in the United States (US) will be classified as obese (BMI ≥ 30 kg/m^2^) and nearly 25% will be classified as severely obese (BMI ≥ 35 kg/m^2^) by the year 2030 [26]. Additionally, nearly 70% of primary TKA patients are projected to be obese by the end of the current decade [3]. Although a clear understanding of obesity is still developing, the local and systemic impact of obesity is well documented. Obesity is often identified as a marked risk factor for post-TKA PJI [2, 16].

Despite being a relatively rare complication, PJI and its associated morbidity carry extensive implications from the individual patient level to the health care system level. Projections estimate the US financial burden of PJI following TKA will be over $1 billion by the end of the current decade [15]. Improved understanding of the factors that predispose TKA patients to infection has considerable economic and medical impact.

Although there is agreement regarding the impact of obesity on postoperative infection, the utility of BMI as a proxy for obesity has been fraught with inconsistency. There is momentum for local adiposity to be used to stratify infection risk. Waisbren et al. [25] determined that the magnitude of adiposity was a more precise measure of infection risk for SSI across a spectrum of elective surgical procedures. Based on the current methodology, the majority of the current evidence suggests that BMI is not a reliable predictor for infection following TKA. However, the lack of uniformity of the published data demonstrates the need for further investigation into the methods that may best predict the risk of infection following TKA.

Limitations of the current work should be considered when interpretating the derived conclusions. Inherent weaknesses of systematic reviews include the potential for error when aggregating data compiled by numerous sources. Inconsistencies in adipose measurement techniques are a feasible contributor. Although, the current work was able to group six of the included articles based on radiographic measurement, the techniques varied. Within the aggregate sample, one study comprised 62% of the whole which has the potential to bias the findings. The broad search strategy and narrow inclusion criteria strengthen the specificity of the results. Despite the current analysis being made without quantified results, the qualitative descriptive nature of the current work is consistent with an emerging concept which is likely to become more homogenized with increasing investigation.

## Conclusion

The use of adiposity as a proxy for obesity in preoperative evaluation of TKA patients is an emerging concept. Although limited by heterogeneity, the current literature suggests that local adiposity may be a more reliable predictor for infection than BMI following primary TKA.

## Data Availability

The current data will be made available upon reasonable request.
